# The Treatment of Tuberculous Laryngitis

**Published:** 1907-11-09

**Authors:** 


### Laryngology and Rhinology.

### THE TREATMENT OF TUBERCULOUS LARYNGITIS.

Having described the methods at our disposal for the treatment of tuberculous laryngitis, the most important point now remains to indicate the cases in which active local treatment is advisable. In considering this subject it is necessary to keep the object of our treatment clearly in view, and to decide whether we can hope to cure or to arrest the laryngeal disease, or merely to treat the symptoms and relieve suffering. The two factors which must guide us in any individual case are the state of the pulmonary disease and the laryngeal symptoms, and we can divide our patients into groups corresponding to the results which we may reasonably hope to obtain.

In the first group come those cases in which the disease in the lungs is in an early stage, and is not rapidly progressive or is improving, and where the general health is good, cases in which as far as the lungs are concerned the physician would give a good prognosis. Of such cases one may say that a cure is possible, however extensive the laryngeal affection, and that the less disease is apparent in the lungs the more energetic should our treatment be; usually in these cases the laryngeal lesions consist only of superficial infiltration or ulceration of a vocal cord or inter-arytenoid space, and will react readily to the application of pigments, but if it be more advanced the curette or punch-forceps should be used without delay.

At the other end of the series are the hopeless cases, in which even temporary arrest cannot be expected. These are the cases where there is far advanced or rapidly progressing disease of the lungs, rapid loss of weight, hectic fever, or severe cachexia; cases of miliary tuberculosis, and cases in which the pulmonary disease is chronic, though advanced, if the laryngeal lesions are very extensive. In all these cases treatment must be entirely symptomatic; the routine clipping and cauterisation of the throats of the cachectic subjects of advanced consumption is both useless and cruel, and has tended to bring the entire system of treatment into disrepute. If there be no severe symptoms, only such mild measures as do not distress the patient should be employed; a steam-inhalation of Friar's balsam or a nebuliser containing menthol and camphor in paraffinum liquidum may be used to diminish the discomfort, and warm intra-tracheal injections of a 1 per cent. oily solution of menthol are often useful if properly given. When dysphagia is present anodynes must be prescribed; morphine and cocaine must be cautiously administered by a skilled attendant; they rapidly lose their action, and they have a disastrous effect on the appetite and general health; they should therefore be reserved for the very last stage if all else fails to give relief. Morphine is best given about half an hour before food in the form of an insufflation in doses of $\frac{1}{6}$ to $\frac{1}{2}$ grain diluted with an inert powder; cocaine should be used just before meals as a 5 per cent. spray. The best analgesic for this purpose, however, is orthoform: it is a non-toxic insoluble powder, and may be entrusted to the patient in the form of an insufflation either alone or diluted with an antiseptic dusting powder; the best effect is obtained in half an hour, and lasts for a long time. A modification of Leduc's auto-insufflation is a convenient method of administration; three to five grains of the powder are put into one end of a straight glass tube, the patient places the other end in the mouth, and by a sharp inspiration draws the drug into the larynx. Much may also be done by careful attention to the diet; patients differ much as to what they can swallow with least discomfort. When, however, dysphagia is severe, by far the most efficacious method is to relieve the tension of the swollen parts by means of the punch-forceps. The removal of the infiltrated epiglottis, or clipping a piece from a swollen arytenoid, has a most remarkable effect on the dysphagia, and causes the entire mass to shrink; the operation takes but a few seconds, causes very little pain under cocaine, and can be performed on almost any patient, however advanced the disease. It is extraordinary how quickly and well these wounds heal even when the larynx is extensively ulcerated, and the patient is in the last stage of pulmonary consumption.

Between these two groups come the large series of intermediate cases, where the lung disease is too advanced for complete cure, but where the adverse symptoms of the last group are absent. They are chiefly cases of chronic phthisis, cases with fibrosis or dry cavities, and cases with quiescent disease. It is here that opinions differ most as to the advisability of local treatment; many of them, however, have the prospect of years of life and usefulness but for the laryngeal complication, and this can often be arrested and sometimes cured by local treatment properly applied. Each case must be judged on its merits, and much depends on the extent of the disease, a localised lesion being often curable in cases of comparatively advanced chronic phthisis. One of the chief difficulties is to properly appraise the extent and progress of the pulmonary affection, for in cases of advanced tuberculous laryngitis the physical signs in the chest are often masked to an extraordinary extent. Before any attempt is made to cure the laryngeal mischief by surgical measures the patient should be kept under observation for two or three weeks, and the temperature and body-weight carefully observed. The whole value of the local treatment of laryngeal tuberculosis depends on careful selection of the cases. Very much can be done if only local treatment be begun at the earliest onset of the disease; but, as tuberculous laryngitis often causes no symptoms in its early stage, it follows that inspection of the larynges of all consumptives at regular intervals, and as a matter of routine, is a most important aid to effective treatment.

